# HIV and HCV screening by non-infectious diseases physicians: can we improve testing and hidden infection rates?

**DOI:** 10.3389/fpubh.2023.1136988

**Published:** 2023-06-26

**Authors:** Alejandro G. García-Ruiz de Morales, Javier Martínez-Sanz, María J. Vivancos-Gallego, Matilde Sánchez-Conde, Manuel Vélez-Díaz-Pallarés, Beatriz Romero-Hernández, María Dolores González Vázquez, Carmen María Cano de Luque, Ander González-Sarria, Juan Carlos Galán, Francisco Gea Rodríguez, Santiago Moreno, María Jesús Pérez-Elías

**Affiliations:** ^1^Department of Infectious Diseases, Hospital Universitario Ramón y Cajal and Instituto de Investigación Sanitaria Ramón y Cajal (IRYCIS), Madrid, Spain; ^2^Universidad de Alcalá, Alcalá de Henares, Spain; ^3^CIBER de enfermedades infecciosas (CIBERINFEC), Instituto de Salud Carlos III, Madrid, Spain; ^4^Department of Pharmacy, Hospital Universitario Ramón y Cajal and Instituto Ramón y Cajal de Investigación Sanitaria IRYCIS, Madrid, Spain; ^5^Department of Microbiology, Hospital Universitario Ramón y Cajal and IRYCIS, Madrid, Spain; ^6^CIBER of Epidemiology and Public Health (CIBERESP), Madrid, Spain; ^7^Department of Internal Medicine, Hospital de Braga, Braga, Portugal; ^8^Department of Internal Medicine, Hospital Clínico San Carlos, Madrid, Spain; ^9^Department of Microbiology, Hospital Universitario Príncipe de Asturias, Alcalá de Henares, Spain; ^10^Department of Gastroenterology, Hospital Universitario Ramón y Cajal and IRYCIS, Madrid, Spain

**Keywords:** formative session, hospital intervention, HIV testing, HCV testing, screening, missed opportunities, late diagnosis, diagnostic screening programs

## Abstract

**Background:**

Missed opportunities for Human Immunodeficiency Virus (HIV) and Hepatitis C Virus (HCV) testing remain high. We aimed to ascertain the knowledge of screening guidelines and attitudes of non-infectious disease (ID) hospital physicians and assess the impact of a 1-h session on screening rates and diagnoses.

**Methods:**

This interventional study consisted of a 1-h training session on HIV and HCV epidemiology and testing guidelines for non-ID physicians. Pre-and post-session questionnaires compared the knowledge of the guidelines and attitudes toward screening before and after the session. Rates of screening and diagnoses were compared in three 6 months periods: before, immediately after, and 24 months ±4 after the session.

**Results:**

A total of 345 physicians from 31 departments participated in these sessions. Before the session, 19.9% (28% medical, 8% surgical) and 17.9% (30% medical, 2.7% surgical) were aware of HIV and HCV testing guidelines, respectively. The willingness to routinely test increased from 5.6 to 22%, whereas not ordering tests decreased from 34.1 to 2.4%. HIV screening rates significantly increased by 20% after the session (7.7 vs. 9.3 tests per 103 patients; *p* < 0.001), and the effect persisted until the long-term period. The HIV diagnosis rate increased globally (3.6 vs. 5.2 HIV diagnoses per 105 patients; *p* = 0.157), mainly because of medical services (4.7 vs. 7.7 per 105 patients; *p* = 0.082). The HCV screening rate increased significantly immediately and in the long term only in medical services (15.7 and 13.6%, respectively). The new active HCV infection rates increased immediately and declined steeply thereafter.

**Conclusion:**

A short session for non-ID physicians can improve HIV/HCV screening, increase diagnosis, and contribute to disease elimination.

## 1. Background

Human Immunodeficiency Virus (HIV) and Hepatitis C Virus (HCV) infections are two of the leading causes of disease worldwide, and are associated with high morbidity and mortality (1, 2). Delayed diagnosis of both infections poses a significant challenge to public health. It is associated with a higher probability of long-term sequelae, increased mortality, and greater risk of onward transmission (3–6).

Current HIV and HCV treatments are simple, safe, and highly effective in suppressing viral replication and preventing morbidity, mortality, and transmission in HIV (3, 5), curing HCV infection, reducing long-term liver sequelae, and preventing cirrhosis and hepatocellular carcinoma (6). The sooner both treatments are started, the lesser the long-term consequences (4). Despite this, screening of asymptomatic patients is poorly implemented (7–9), and testing in many European countries remains guided by risk behaviors and limited to populations with a known high prevalence of both infections (7, 9).

HIV and HCV testing can be performed by any physician both in hospitals and primary care facilities. Missed testing opportunities in both locations remain high (10–16), although some primary care educational interventions have improved screening rates (17, 18). Nevertheless, among hospital noninfectious disease (ID) specialists, reports on such interventions are scarce.

Given the similarity in risk factors and the population affected by both infections, we aimed to describe the knowledge on screening policies and the current routine HIV and HCV screening practices of non-ID physicians in a tertiary hospital and to assess the impact of a 1-h training session on screening rates of both infections.

## 2. Materials and methods

We conducted an interventional study at Ramón y Cajal Hospital in Madrid, Spain, a tertiary hospital with 900 beds and 32 medical and surgical departments covering a population of approximately 600,000 inhabitants. From February to November 2019, five physicians (four ID specialists and one gastroenterologist) provided a non-mandatory 1-h training session on HIV, HCV, and other sexually transmitted infections, including epidemiology, risk factors, clinical aspects, and screening, to the other 31 hospital departments (17 medical and 14 surgical, Supplementary Table S1).

First, a brief questionnaire was used before training (Supplementary Tables S2, S3) to evaluate previous attitudes toward screening. After the training, a second questionnaire was used to evaluate the immediate effect of the session on participants’ expressed future attitudes toward testing. We performed a before-after comparative analysis of the responses to both questionnaires. Second, we compared the absolute number of HIV and HCV tests requested, the screening rate per 1,000 patients attended, and the new HIV and HCV diagnoses and positivity rates per 100,000 patients attended in the 6 months before and after the training, globally, and for each department. Finally, we analyzed the long-term effect of the intervention by comparing the rates to those obtained 21 to 28 months after the training sessions. We excluded serologies from known HIV-positive patients and follow-up serologies from HCV-untreated patients.

The number of patients attended per period and department was obtained from the hospital’s electronic records. In contrast, the number of ordered tests, positive tests, and new diagnoses was obtained from the records of the Microbiology, Infectious Diseases, and Gastroenterology departments.

The follow-up for each department in the pre-, post-training, and long-term periods was expected to be 180 days. Nevertheless, due to the impact of the Coronavirus Disease 2019 (COVID-19) pandemic, the normal workflow of each department has changed. To avoid possible biases due to modifications in the number of attended patients and serologies requested by each specific department, we decided to censor follow-up for those departments that had not completed their post-training observation period by March 14, 2020. For each department, the number of evaluated days before the session was matched to that in the post-training and long-term periods (Supplementary Table S1).

We used medians and interquartile ranges (IQR) for descriptive analysis, Fisher’s exact test to compare categorical variables, and Wilcoxon’s signed-rank test to compare paired quantitative variables. All contrasts were bilateral, and *p* values <0.05 were considered statistically significant. Statistical analysis was performed using Stata® 17.0 software (Stata Corp-LP, College Station, TX, United States) and Microsoft® Excel 16.67 software (Microsoft Corp., Redmond, WS United States,). GraphPad Prism 9 (GraphPad Software Inc.) was used to generate the figures.

## 3. Results

The training sessions included 345 physicians from 31 hospital departments, with a median of 16 physicians per department (IQR 6–23). They were mainly women (59.3%) and under 40 years of age (67.3%; only 9.8% were above 55 years old).

### 3.1. Training sessions and questionnaire results

According to the pre-training questionnaire, 19.9% (28.0% medical, 8.0% surgical; *p* < 0.001) and 17.9% (30.0% medical, 2.7% surgical; *p* < 0.001) of the participants were aware of HIV and HCV testing guidelines, respectively. When asked whether they ordered these tests during their usual practice, 5.6% claimed to ask for them routinely (9.5% medical, 0.7% surgical; *p* < 0.001), 60.4% if there were identifiable risk factors and/or indicator conditions (72.6% medical, 45.1% surgical; *p* < 0.001), and 34.1% never ordered them (17.9% medical, 54.3% surgical; *p* < 0.001; Supplementary Table S2).

Regarding the post-training questionnaire, we observed a significant increase in physicians who showed a positive attitude toward routinely asking for screening tests (5.6% vs. 22.0%; *p* < 0.001), and a significant decline in those who did not consider ordering a test (34.1% vs. 2.4%; p < 0.001). In addition, 98.2% of the participants (97.9% medical, 98.6% surgical, *p* = 0.485) considered the training session and potential availability of a tool to guide HIV and HCV testing. Supplementary Tables S2, S3 show the data detailed by medical and surgical specialty pre-and post-training.

### 3.2. HIV and HCV screening.

Supplementary Table S1 shows the dates of the training sessions and the number of patients who attended each department during the corresponding observation period.

**Figure 1:**
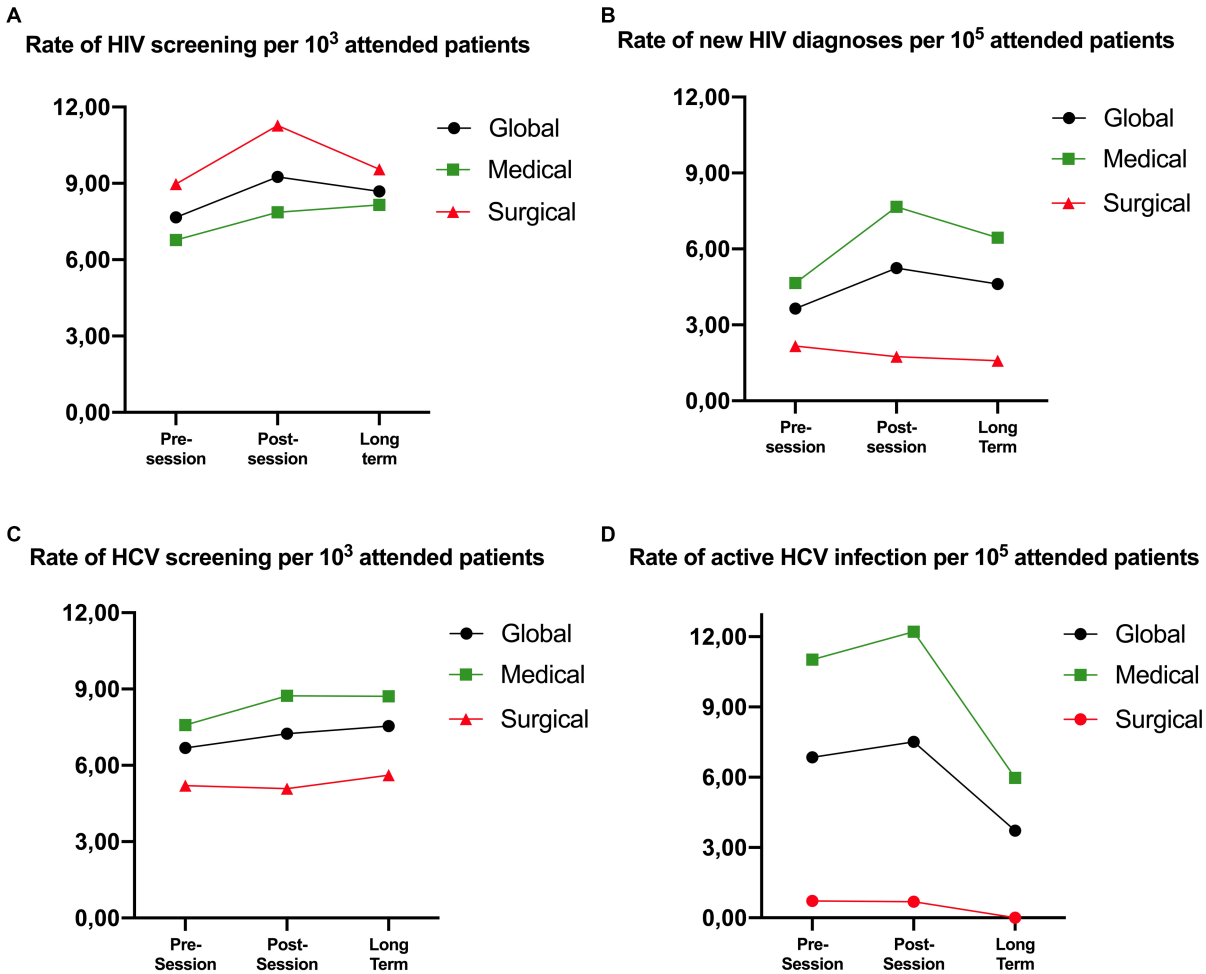
Evolution of the global screening rate and active infection globally, in medical and surgical departments. The figure shows the evolution of the rate of screening **(A,C)** and active infection **(B,D)** for HIV **(A,B)** and HCV **(C,D)** in the pre-, post-session and long-term periods. Each sub-figure has a representation of the global rates broken down by medical and surgical departments. * Active HCV infection is defined as a positive HCV antigen.

5,257 pre-training and 6,532 post-training HIV serologies were requested (7.7 vs. 9.3 tests per 103 attended patients; *p* < 0.001). This increase was significant both in medical and surgical departments (6.8 vs. 7.9 tests per 10^3^ attended patients, *p* < 0.001; and 9.0 vs. 11.3, *p* < 0.001 respectively; Figure 1; Table 1), although only driven by six medical departments and just one surgical department (Table S4a). Overall, we found 25 pre-training and 37 post-training positive tests (3.6 vs. 5.2 HIV diagnoses per 10^5^ attended patients; *p* = 0.157), but this increase was only seen in medical departments (19 vs. 32, corresponding to 4.7 vs. 7.7 diagnoses per 10^5^ attended patients; *p* = 0.082; Figure 1; Table 1). These new diagnoses were concentrated in the Emergency Department (5.0 vs. 12.0 diagnoses per 10^5^ attended patients; *p* = 0.137), Preventive Medicine Department (99.8 vs. 290.8; *p* = 0.161), and Nephrology (12.2 vs. 38.1; *p* = 0.133) (Supplementary Table S4b).

**Table 1:** HIV and HCV screening and active infection rates globally and split by medical and surgical departments.

Departments		Ordered tests/10^3^ attended patients				Positive tests (HIV) or new active HCV infections /10^5^ attended patients					
		Before Training	After Training	*p value*	Long-term	p value^*^	Before Training	After Training	*p value*	Long-term	p value^*^
**HIV**	Globally	7.66	9.25	<0.001	8.68	<0.001	3.64	5.24	0.157	4.61	0.377
	Medical	6.77	7.86	<0.001	8.15	<0.001	4.65	7.66	0.082	6.45	0.274
	Surgical	8.97	11.27	<0.001	9.55	0.024	2.16	1.74	0.718	1.58	0.629
**HCV**	Globally	6.62	7.24	<0.001	7.54	<0.001	6.85	7.51	0.646	3.72	0.012
	Medical	7.58	8.73	<0.001	8.71	<0.001	11.02	12.21	0.616	5.97	0.013
	Surgical	5.20	5.08	0.534	5.61	0.030	0.72	0.69	0.971	0	0.177

New active HCV infections are considered when HCV antigen is positive.

* value of *p* long-term vs. before training.

For HCV, 4,541 pre-training and 5,113 post-training serologies were requested (6.6 vs. 7.2 tests per 10^3^ attended patients, *p* < 0.001) with a significant increase in the screening rate in medical departments (+15.2%, p < 0.001) but not in surgical departments (*p* = 0.534; Figure 1; Table 1). Globally, HCV prevalence among the serologies requested was 3.3% before and 3.4% after training. A total of 47 pre-training and 53 post-training active HCV infections were diagnosed (positive HCV antigen), with a non-significant 9.6% increase in the active infection rate after the training (global active infection rate 6.9 vs. 7.5 per 10^5^ attended patients, *p* = 0.646; Figure 1; Table 1).

### 3.3. Long-term effect of the intervention.

A total of 5,832 HIV tests were requested in the long-term period with a global increase of 13.5% as compared to the pre-training period (7.7 vs. 8.7 tests per 10^3^ attended patients; *p* < 0.001) (1.39 and 0.58 tests increase per 10^3^ patients, respectively, in medical and surgical departments; p < 0.001 and 0.019, respectively; Figure 1; Table 1). We observed a non-significant 26.7% increase in HIV diagnoses (3.6 vs. 4.6 diagnoses per 10^5^ attended patients; *p* = 0.377) driven only by medical departments (Figure 1; Table 1).

Overall, physicians increased their HCV screening rate by 13% as compared to the pre-session period (6.6 vs. 7.5 tests per 10^3^ attended patients; *p* < 0.001; Figure 1, Table 1), with a non-significant increase of 10.9% in positive HCV serologies (22.1 vs. 24.6 per 10^5^ attended patients; *p* = 0.284) (Table S5b), and a significant decrease in the rates of HCV active infection found (6.9 vs. 3.7 diagnoses per 10^5^ attended patients; *p* = 0.012; Figure 1; Table 1). Detailed screening rates and diagnoses stratified by department can be found in the Supplementary Tables S4A,B for HIV and S5a, S5b, and S5c for HCV.

## 4. Discussion

Implementing a one-hour teaching intervention for non-ID specialists in this tertiary hospital immediately increased the overall HIV and HCV testing rates, with a more significant effect seen in medical departments. This effect remained evident until at least 2 years after the training, although the impact in surgical departments was milder and wore out faster. This type of intervention has the potential to be widely implemented, as its duration corresponds with the formative sessions of most departments, covers direct and simple concepts, and can be adapted to each specific service. The questionnaire results showed a significant lack of knowledge regarding the testing guidelines.

The rates and absolute number of new HIV diagnoses were higher immediately and 2 years after the intervention. This increase, albeit moderate, has the potential to reduce the number of hidden HIV infections. The HCV diagnostic rates were similar before and after the session but steeply declined 2 years later.

Universal opt-out HIV testing programs offered to all general medical admissions in a hospital have proven effective in improving HIV diagnosis in several studies (19). Formative strategies have proven to be helpful in primary care (17, 18, 20). However, data on these strategies in hospital settings are scarce. We herein show that immediate rates of screening can rise after a simple 1-h training on screening guidelines and general sexually transmitted infections, and that hospital healthcare workers who take part in the sessions improve their willingness to screen for HIV/HCV both routinely and guided by risk factors.

Interestingly, significant differences were observed between the medical and surgical departments regarding their initial knowledge of the guidelines and willingness to order tests. These results could be explained by the fact that, in our hospital, preoperative tests are performed by anesthesiologists, while surgeons frequently rely on them for pre-surgical blood tests. Despite this, our training session greatly impacted the surgeons’ attitudes toward HIV and HCV testing. However, excluding gynecologists and dermatologists, screening and diagnostic rates did not improve, and surgeons’ diagnostic rates remained very low throughout the observed periods. We do not have any evidence on the impact of this type of strategy in surgical departments to compare our results. Since anesthesiologists always have contact with surgical patients before surgery, focusing on improving their screening and diagnostic rates, both with formative sessions and including the tests routinely in preoperative protocols (21), could be more valuable than improving the rates for surgeons.

Although a clear increase was observed in HIV and HCV screening rates, the growth in the rates of active infection found after training was mild, probably due to the low prevalence of infection in the observed population. Nevertheless, HCV screening reached a maximum during the post-training period and declined sharply thereafter. This could be due to the overall low number of diagnoses but also to the decline in the global number of HCV infections in Madrid Region since 2017 and, more importantly, since 2019 (22, 23), Reporting delay due to the COVID-19 pandemic could explain this substantial reduction in the rate of active HCV infection. Moreover, this could also be due to the availability of direct antivirals. These treatments were included by the Spanish Health System in 2014 with many restrictions (24), but by 2017, the use of pangenotypic treatments was extended to any infected patient (25, 26), making Spain the leading country in HCV-treated patients per million inhabitants in 2022, and one of the top contenders to achieve HCV eradication in the present decade (27).

Spain managed to reach two of (Joint United Nations Programme on HIV/AIDS (UNAIDS) 90% goals in 2020, coming short only in the diagnostic goal (28, 29); Therefore, different strategies should be pursued to achieve the 95% goals set by UNAIDS in 2030. Given the latest estimations, Madrid’s current HIV prevalence is 0.33% (30), and 13% of the patients remain undiagnosed (29); hence, we can estimate that approximately 250 patients could remain HIV undiagnosed in our health area. Our session on its own managed to increase the rate of HIV diagnoses 1.6 per 105 attended patients), which would mean diagnosing eight new patients in our assigned population. The results of this easy and reproducible intervention would allow us to diagnose more than 3% of the undiagnosed patients in our population and help progressively reduce the burden of hidden HIV infection. Given that HIV screening is cost-effective, the increase in the number of serologies requested is not expected to have a negative impact on the health system.

Thus far, no strategies based on training sessions to hospital physicians had demonstrated effectiveness up to now (19, 31, 32). Our results demonstrate an easy and feasible way to increase HIV and HCV screening rates in the population. Combined with other strategies, they could be helpful in improving the rate of undiagnosed patients and avoiding missed opportunities for HIV and HCV testing at hospital facilities (13–15); the same way they have proven to be effective at community and primary care levels (17, 18).

Despite the hardwearing effect that can be seen in our study, the impact of a new session, months after the first session, focusing on the lack of each service, could be used to assess whether periodic training could help to further reduce the burden of hidden infections.

Our study has some limitations. First, the COVID-19 pandemic struck before the post-session observation was completed in some departments, so we were obliged to reduce the pre-and post-observation periods in some departments; however, it also disturbed the standard workflow up to the present day (33), and although mild, some effects could still be seen during the long-term period. Second, the positive impact of the training is not consistent between medical and surgical departments, as in the latter, the impact of the intervention is negligible, and further interventions must be implemented to improve screening rates. In addition, the absolute increases in screening rates and active infections are low, and it could be argued that its contribution to effectively decreasing the rate of hidden infections is limited. Although medical training in Spain is homogeneous, we were unable to collect data on participants’ prior HIV-specific training. Nevertheless, we demonstrate that a simple intervention can improve screening rates in several hospital departments and can be used to reduce the burden of hidden HIV and HCV diseases. Further multicenter studies should be conducted to assess whether these interventions can be extrapolated to other hospitals, and whether the intervention should be repeated periodically to consolidate its long-term effect.

In conclusion, a formative session for hospital physicians can improve HIV/HCV screening and diagnostic rates and therefore help eliminate both infections, although other strategies should also be explored. Different strategies to tackle surgeons’ and other medical specialties’ reluctance to test must be added to the formative session to automatize and personalize the different strategies in each department. More extensive multicenter studies are needed to confirm these results.

## Data availability statement

The raw data supporting the conclusions of this article will be made available by the authors, without undue reservation.

## Ethics statement

The studies involving human participants were reviewed and approved by Ethics Committee at University Hospital Ramón y Cajal (ceic.hrc@salud.madrid.org) with ID number 001-23. Written informed consent for participation was not required for this study in accordance with the national legislation and the institutional requirements.

## Author contributions

MV-G, SM, and MP-E designed the study. AG-R and JM-S performed the research and the statistical analyses. AG-R, JM-S, MV-G, SM, and MP-E wrote the original draft. JM-S, MV-G, MS-C, BR-H, FR, and MP-E gave the formative sessions. MV-D-P, MDV, CL, AG-S, and JG helped with the data collection. All authors contributed to the article and approved the submitted version.

## Funding

This study has been funded by unrestricted grants 2018/0371, 2021/0193 and 2019/034 and by Instituto de Salud Carlos III (ISCIII) through the project PI22/01878, the Spanish Ministry of Science and Innovation and co-funded by the European Union.

## Conflict of interest

AG-R reports personal fees for educational events and non-financial support, including support for attending meetings and/or travel from ViiV Healthcare, Gilead Sciences, Merck Sharp & Dohme and Abbvie, outside the submitted work. JM-S reports personal fees for presentations or educational events and non-financial support, including support for attending meetings and/or travel, from ViiV Healthcare, Janssen Cilag, Gilead Sciences, Merck Sharp & Dohme and Abbvie outside the submitted work. MV-G has received honoraria (grants and personal fees) as a speaker in educational programs sponsored by ViiV, and Gilead; and has received support (registration, travel assistance) for expert courses and congresses by MSD and ViiV. BR-H has received funding to attend conferences from Gilead Sciences and obtained a financed project from Roche. SM has been involved in speaking activities and received grants for research from Gilead Sciences, Janssen Cilag, Merck, Sharp & Dohme and ViiV Healthcare. MP-E has received funding to attend conferences, educational activities or advisory, as well as scholarships from the pharmaceutical companies Gilead Sciences, Janssen, Abbvie, MSD, and ViiV. JG has received funding to attend conferences, educational activities or advisory, from the pharmaceutical companies Gilead Sciences, Abbvie, Abbott, MSD and Roche.

The remaining authors declare that the research was conducted in the absence of any commercial or financial relationships that could be construed as a potential conflict of interest.

## Publisher’s note

All claims expressed in this article are solely those of the authors and do not necessarily represent those of their affiliated organizations, or those of the publisher, the editors and the reviewers. Any product that may be evaluated in this article, or claim that may be made by its manufacturer, is not guaranteed or endorsed by the publisher.

## Abbreviations

HCV: Hepatitis C virus
HIV: Human Immunodeficiency Virus
ID: infectious diseases
COVID-19: Coronavirus Disease 2019
IQR: interquartile range
vs: versus
UNAIDS: Joint United Nations Programme on HIV/AIDS
