# Supplementary material for: HIV and HCV screening by non-infectious diseases physicians: can we improve testing and hidden infection rates?
Source: Front Public Health. 2023 Jun 26;11:1136988. doi: 10.3389/fpubh.2023.1136988 (PMC10332317; doi:10.3389/fpubh.2023.1136988)
Supplement: Supplementary file 1 [file Table_1.docx]

Supplementary Material

**HIV and HCV screening by non-infectious diseases physicians: can we improve testing and hidden infection rates?**

**Alejandro G. García-Ruiz de Morales^1,2,3*^, Javier Martínez-Sanz^1,3*^, María Jesús Vivancos^1,3^, Matilde Sánchez-Conde^1,3^, Manuel Vélez-Díaz-Pallarés^4^, Beatriz Romero-Hernández^6,7^, María Dolores González Vázquez^8^, Carmen María Cano de Luque^9^, Ander González-Sarria^10^, Juan Carlos Galán^6,7^, Francisco Gea Rodríguez^5^, Santiago Moreno^1,2,3^**^†^**, María Jesús Pérez-Elías^1,3^**^†^**.**

*** Corresponding authors:**Alejandro G. García-Ruiz de Morales. HIV unit. Infectious Diseases Department. Hospital Universitario Ramón y Cajal. Carretera de Colmenar Km 9.1. Madrid 28034. Spain. E-mail: [alejandrogarciagarcia@gmail.com](mailto:alejandrogarciagarcia@gmail.com). Phone: +34 913368108. Fax: +34 913368238

Javier Martínez Sanz. HIV unit. Infectious Diseases Department. Hospital Universitario Ramón y Cajal. Carretera de Colmenar Km 9.1. Madrid 28034. Spain. E-mail: [javier.martinez.sanz@salud.madrid.org](mailto:javier.martinez.sanz@salud.madrid.org). Phone: +34 913368108. Fax: +34 913368238

**TABLE S1.** Date of session, number of observation days pre-, post-session, and long-term, and number of attended patients per period and department.

|  | Date of the Session | Number of observed days | Attended patients pre-session | Attended patients post-session | Long-term start date* | Attended patients long-term |
| --- | --- | --- | --- | --- | --- | --- |
| **Medical Departments** |  | | | |  |  |
| Emergency Department | 8/4/19 | 180 | 79223 | 74884 | 4/7/21 | 74721 |
| Radiotherapeutic Oncology | 9/4/19 | 180 | 13180 | 10005 | 4/7/21 | 22994 |
| Pediatrics | 25/4/19 | 180 | 25269 | 24029 | 4/7/21 | 27602 |
| Gastroenterology | 13/5/19 | 180 | 47736 | 48215 | 4/7/21 | 44829 |
| Neurology | 14/5/19 | 180 | 23196 | 21694 | 4/7/21 | 22231 |
| Endocrinology | 28/5/19 | 180 | 25437 | 29266 | 4/7/21 | 28852 |
| Intensive Care | 12/6/19 | 180 | 315 | 275 | 4/7/21 | 436 |
| Preventive Medicine | 19/6/19 | 180 | 2005 | 2407 | 4/7/21 | 4917 |
| Immunology | 25/6/19 | 180 | 508 | 580 | 4/7/21 | 730 |
| Internal Medicine | 25/6/19 | 180 | 16087 | 14218 | 4/7/21 | 15951 |
| Medical Oncology | 12/9/19 | 180 | 38678 | 41903 | 4/7/21 | 32266 |
| Pneumonology | 13/9/19 | 180 | 24493 | 27610 | 4/7/21 | 31820 |
| Allergy | 26/9/19 | 170 | 18062 | 20258 | 14/7/21 | 20314 |
| Nephrology | 15/10/19 | 151 | 16442 | 18369 | 2/8/21 | 15631 |
| Psychiatry | 16/10/19 | 150 | 11878 | 11588 | 3/8/21 | 7263 |
| Cardiology | 21/10/19 | 145 | 51704 | 57277 | 8/8/21 | 51383 |
| Rheumatology | 14/11/19 | 121 | 14198 | 15229 | 1/9/21 | 16818 |
| **Surgical Departments** |  | | | |  |  |
| Anesthesiology | 22/2/19 | 180 | 13399 | 13378 | 4/7/21 | 12326 |
| Ophthalmology | 12/3/19 | 180 | 65292 | 64794 | 4/7/21 | 61043 |
| Gynecology | 15/3/19 | 180 | 39196 | 38113 | 4/7/21 | 36531 |
| Vascular Surgery | 29/3/19 | 180 | 12639 | 11378 | 4/7/21 | 8435 |
| Dermatology | 4/4/19 | 180 | 29530 | 29773 | 4/7/21 | 22746 |
| Plastic Surgery | 21/5/19 | 180 | 8658 | 8814 | 4/7/21 | 7356 |
| Thoracic Surgery | 27/9/19 | 169 | 1527 | 1624 | 15/7/21 | 1734 |
| Maxillofacial Surgery | 27/9/19 | 169 | 11123 | 12042 | 15/7/21 | 9126 |
| General Surgery | 4/10/19 | 162 | 16522 | 18743 | 22/7/21 | 15551 |
| Neurosurgery | 15/10/19 | 151 | 3335 | 3607 | 2/8/21 | 2734 |
| Otorhinolaryngology | 22/10/19 | 144 | 20746 | 24575 | 9/8/21 | 19323 |
| Traumatology | 23/10/19 | 143 | 34849 | 38275 | 10/8/21 | 33441 |
| Cardiac Surgery | 15/11/19 | 120 | 1250 | 1592 | 2/9/21 | 1651 |
| Urology | 21/11/19 | 114 | 19944 | 21503 | 8/9/21 | 21299 |

* Long-term end date was 12/31/21 for all departments

**TABLE S2. Questions in the pre-session questionnaire and answers by medical and surgical departments.**

| Pre-session questionnaire | | | | |
| --- | --- | --- | --- | --- |
|  | Total | Medical | Surgical | p-value |
| *Do you know the HIV screening guidelines?*   - No - Yes | 80.9%  19.9% | 71.9%  28.1% | 92.2%  7.8% | p<0.001 |
| *Do you know the HCV screening guidelines?*   - No - Yes | 82.1%  17.9% | 70.0%  30.0% | 97.4%  2.7% | p<0.001 |
| *Do you usually screen for HIV/HCV?*   - Always - If evident risk present - If indicator conditions present - If both of the previous present - I don’t normally order them | 5.6%  11.1%  6.1%  43.2%  34.1% | 9.5%  11.6%  4.7%  56.3%  17.9% | 0.7%  10.5%  7.8%  26.8%  54.3% | p<0.001 |
| *Conditions that guide you to order a HIV/HCV test*   - Patient’s request - External appearance - I use external tools or scores - I follow available guidelines - It’s not on my priorities during my practice | 8.5%  16.3%  8.5%  23.9%  42.9% | 9.0%  16.8%  10.0%  36.3%  27.9% | 7.8%  15.7%  6.5%  8.5%  61.4% | p<0.001 |
| *Do you normally think on asymptomatic STIs?*   - Frequently - Sometimes - Only if very evident | 18.1%  41.7%  40.2% | 16.3%  46.8%  36.8% | 20.3%  35.3%  44.4% | p=0.097 |
| *Do you think a tool to guide you on HIV screening could be useful?*   - No - Yes | 1.5%  98.5% | 1.1%  99.0% | 2.0%  98.0% | p=0.485 |

**TABLE S3. Questions in the post-session questionnaire and answers by medical and surgical departments.**

| Post-session Questionnaire | | | | |
| --- | --- | --- | --- | --- |
|  | Total | Medical | Surgical | **p-value** |
| *Will you order tests in your practice from now on?*   - Always - If evident risk present - If indicator conditions present - If both of the previous present - I won’t normally order them | 22.1%  9.6%  2.7%  62.3%  2.4% | 26.8%  8.4%  1.6%  60.5%  2.6% | 15.9%  9.6%  4.1%  66.9%  2.1% | p=0.102 |
| *Conditions that will guide you to order a HIV/HCV test*   - Patient’s request - External appearance - I use external tools or scores - I follow available guidelines - It won’t be on my priorities during my practice | 7.2%  8.1%  35.2%  46.0%  3.6% | 7.9%  5.8%  35.8%  48.9%  1.6% | 6.2%  11.0%  34.5%  42.1%  6.2% | p=0.063 |
| *Will you think on asymptomatic STIs?*   - Frequently - Sometimes - Only if very evident | 57.6%  33.4%  9.0% | 61.6%  31.1%  7.4% | 52.4%  36.6%  11.0% | p=0.204 |
| *Do you think the session was useful to improve your HIV/HCV screening?*   - No - Yes | 1.8%  98.2% | 2.1%  97.9% | 1.4%  98.6% | p=0.620 |

***Table S4a.*** *HIV screening rate per 10^3^ attended patients per department in pre-, post-session, and long-term periods.*

| Departments | HIV tests/10^3^ attended patients | | | | |
| --- | --- | --- | --- | --- | --- |
|  | Before Training | After  Training | *p*-value | Long-term | *p*-value* |
| **Globally: Medical + Surgical** | 7.66 | 9.25 | <0.001 | 8.68 | <0.001 |
| **Medical** | 6.77 | 7.86 | <0.001 | 8.15 | <0.001 |
| Emergency Department | 3.32 | 4.30 | **0.002** | 7.31 | <0.001 |
| Radiotherapeutic Oncology | 0.46 | 0.20 | 0.300 | 0.30 | 0.466 |
| Pediatrics | 0.99 | 0.96 | 0.909 | 1.12 | 0.637 |
| Gastroenterology | 8.32 | 10.14 | **0.003** | 7.32 | 0.085 |
| Neurology | 12.16 | 14.47 | 0.032 | 14.53 | 0.027 |
| Endocrinology | 8.41 | 9.67 | 0.122 | 2.74 | <0.001 |
| Intensive Care | 50.79 | 145.45 | **<0.001** | 149.08 | <0.001 |
| Preventive Medicine | 33.42 | 65.23 | **<0.001** | 24.41 | 0.036 |
| Immunology | 21.65 | 3.45 | 0.006 | 4.11 | 0.004 |
| Internal Medicine | 26.11 | 29.68 | **0.059** | 40.25 | <0.001 |
| Medical Oncology | 6.21 | 6.13 | 0.896 | 8.49 | <0.001 |
| Pneumonology | 4.57 | 4.45 | 0.841 | 6.29 | 0.007 |
| Allergy | 1.05 | 1.33 | 0.428 | 1.48 | 0.245 |
| Nephrology | 28.89 | 32.12 | **0.080** | 29.75 | 0.648 |
| Psychiatry | 6.48 | 5.95 | 0.607 | 9.64 | 0.015 |
| Cardiology | 0.77 | 0.72 | 0.727 | 1.03 | 0.168 |
| Rheumatology | 6.97 | 8.01 | 0.303 | 10.52 | 0.001 |
| **Surgical** | 8.97 | 11.27 | <0.001 | 9.55 | 0.024 |
| Anesthesiology | 4.48 | 4.41 | 0.934 | 4.79 | 0.715 |
| Ophthalmology | 0.18 | 0.32 | 0.112 | 0.15 | 0.616 |
| Gynecology | 48.58 | 68.53 | **<0.001** | 49.08 | 0.747 |
| Vascular Surgery | 1.11 | 1.93 | 0.099 | 1.54 | 0.389 |
| Dermatology | 13.17 | 14.07 | 0.345 | 20.31 | <0.001 |
| Plastic Surgery | 0.69 | 0.23 | 0.160 | 0.41 | 0.448 |
| Thoracic Surgery | 0.65 | 1.23 | 0.600 | 0.58 | 0.928 |
| Maxillofacial Surgery | 0.99 | 1.25 | 0.560 | 1.86 | 0.096 |
| General Surgery | 1.88 | 1.97 | 0.835 | 1.35 | 0.242 |
| Neurosurgery | 0.30 | 0.55 | 0.610 | 1.10 | 0.228 |
| Otorhinolaryngology | 0.87 | 0.61 | 0.312 | 0.52 | 0.185 |
| Traumatology | 0.89 | 0.60 | 0.151 | 0.30 | 0.002 |
| Cardiac Surgery | 0 | 0.63 | 0.376 | 0.00 | - |
| Urology | 0.80 | 0.88 | 0.901 | 0.85 | 0.880 |

* p-value long-term vs. before training

***Table S4b.*** *New HIV diagnoses rate per 10^5^ attended patients per department in pre-, post-session, and long-term periods.*

| Departments | HIV positive tests/10^5^ attended patients | | | | |
| --- | --- | --- | --- | --- | --- |
|  | Before Training | After  Training | *p*-value | Long-term | *p*-value* |
| **Globally: Medical + Surgical** | 3.64 | 5.24 | 0.157 | 4.61 | 0.377 |
| **Medical** | 4.65 | 7.66 | 0.082 | 6.45 | 0.274 |
| Emergency Department | 5.05 | 12.02 | 0.137 | 10.71 | 0.209 |
| Radiotherapeutic Oncology | 0 | 0 | - | 0 | - |
| Pediatrics | 0 | 0 | - | 0 | - |
| Gastroenterology | 2.10 | 2.07 | 0.994 | 2.23 | 0.965 |
| Neurology | 8.62 | 9.22 | 0.947 | 0 | 0.166 |
| Endocrinology | 11.79 | 17.09 | 0.610 | 3.47 | 0.259 |
| Intensive Care | 0 | 0 | - | 0 | - |
| Preventive Medicine | 99.75 | 290.82 | 0.161 | 183.04 | 0.430 |
| Immunology | 0 | 0 | - | 0 | - |
| Internal Medicine | 12.43 | 0 | 0.184 | 0 | 0.159 |
| Medical Oncology | 0 | 0 | - | 6.20 | 0.122 |
| Pneumonology | 4.08 | 3.62 | 0.932 | 3.14 | 0.853 |
| Allergy | 5.54 |  | 0.290 | 4.92 | 0.934 |
| Nephrology | 12.16 | 38.11 | 0.133 | 25.59 | 0.380 |
| Psychiatry | 0 | 0 | - | 0 | - |
| Cardiology | 0 | 0 | - | 0 | - |
| Rheumatology | 7.04 | 0 | 0.300 | 0 | 0.276 |
| **Surgical** | 2.16 | 1.74 | 0.718 | 1.58 | 0.629 |
| Anesthesiology | 0 | 0 | - | 0 | - |
| Ophthalmology | 0 | 0 | - | 0 | - |
| Gynecology | 0 | 2.62 | 0.311 | 2.74 | 0.300 |
| Vascular Surgery | 0 | 0 | - | 23.71 | 0.083 |
| Dermatology | 16.93 | 13.44 | 0.730 | 4.40 | 0.185 |
| Plastic Surgery | 0 | 0 | - | 0 | - |
| Thoracic Surgery | 0 | 0 | - | 0 | - |
| Maxillofacial Surgery | 0 | 0 | - | 0 | - |
| General Surgery | 0 | 0 | - | 0 | - |
| Neurosurgery | 0 | 0 | - | 0 | - |
| Otorhinolaryngology | 0 | 0 | - | 0 | - |
| Traumatology | 2.87 | 0 | 0.295 | 0 | 0.331 |
| Cardiac Surgery | 0 | 0 | - | 0 | - |
| Urology | 0 | 0 | - | 0 | - |

* p-value long-term vs. before training

***Table S5a.*** *HCV screening rate per 10^3^ attended patients in pre-, post-session, and long-term periods.*

| Departments | HCV tests/10^3^ attended patients | | | | |
| --- | --- | --- | --- | --- | --- |
|  | Before Training | After  Training | *p*-value | Long-term | *p*-value* |
| **Globally: Medical + Surgical** | 6.62 | 7.24 | <0.001 | 7.54 | <0.001 |
| **Medical** | 7.58 | 8.73 | <0.001 | 8.71 | <0.001 |
| Emergency Department | 3.08 | 3.91 | 0.006 | 6.95 | <0.001 |
| Radiotherapeutic Oncology | 0.46 | 0.30 | 0.552 | 0.00 | 0.001 |
| Pediatrics | 1.23 | 1.12 | 0.738 | 1.09 | 0.636 |
| Gastroenterology | 19.48 | 21.76 | 0.013 | 12.78 | <0.001 |
| Neurology | 5.04 | 5.30 | 0.704 | 5.17 | 0.847 |
| Endocrinology | 8.37 | 9.43 | 0.190 | 2.53 | <0.001 |
| Intensive Care | 38.10 | 87.27 | 0.013 | 139.91 | <0.001 |
| Preventive Medicine | 28.93 | 52.76 | <0.001 | 24.61 | 0.304 |
| Immunology | 15.75 | 0 | 0.002 | 5.48 | 0.070 |
| Internal Medicine | 22.50 | 27.85 | 0.003 | 41.06 | <0.001 |
| Medical Oncology | 6.64 | 6.16 | 0.386 | 9.14 | <0.001 |
| Pneumonology | 2.12 | 1.77 | 0.367 | 2.36 | 0.562 |
| Allergy | 1.49 | 2.37 | 0.053 | 2.95 | 0.003 |
| Nephrology | 19.16 | 26.08 | <0.001 | 31.41 | <0.001 |
| Psychiatry | 5.22 | 5.26 | 0.962 | 8.95 | 0.002 |
| Cardiology | 0.33 | 0.44 | 0.366 | 1.11 | <0.001 |
| Rheumatology | 27.05 | 27.45 | 0.833 | 26.88 | 0.927 |
| **Surgical** | 5.20 | 5.08 | 0.534 | 5.61 | 0.030 |
| Anesthesiology | 4.10 | 4.26 | 0.843 | 2.19 | 0.007 |
| Ophthalmology | 0.26 | 0.31 | 0.605 | 0.15 | 0.162 |
| Gynecology | 23.37 | 23.77 | 0.713 | 22.39 | 0.369 |
| Vascular Surgery | 0.95 | 2.11 | 0.020 | 1.54 | 0.221 |
| Dermatology | 12.36 | 12.53 | 0.854 | 20,27 | <0.001 |
| Plastic Surgery | 0.12 | 0 | 0.313 | 0.54 | 0.126 |
| Thoracic Surgery | 1.31 | 0 | 0.145 | 0.58 | 0.491 |
| Maxillofacial Surgery | 0.99 | 1.16 | 0.688 | 1.64 | 0.196 |
| General Surgery | 1.69 | 2.08 | 0.406 | 1.41 | 0.525 |
| Neurosurgery | 0.30 | 0.28 | 0.956 | 1.10 | 0.228 |
| Otorhinolaryngology | 0.53 | 0.41 | 0.543 | 0.47 | 0.773 |
| Traumatology | 0.29 | 0.18 | 0.356 | 0.45 | 0.255 |
| Cardiac Surgery | 0 | 0 | - | 0.00 | - |
| Urology | 0.85 | 0.65 | 0.454 | 1.17 | 0.307 |

* p-value long-term vs. before training

***Table S5b.*** *HCV positive serology rate per 10^5^ attended patients per department in pre-, post-session, and long-term periods.*

| Departments | HCV positive serologies/10^5^ attended patients | | | | |
| --- | --- | --- | --- | --- | --- |
|  | Before Training | After  Training | *p*-value | Long-term | *p*-value* |
| **Globally: Medical + Surgical** | 22.14 | 24.93 | 0.284 | 24.55 | 0.371 |
| **Medical** | 34.03 | 38.77 | 0.259 | 34.87 | 0.848 |
| Emergency Department | 7.57 | 10.68 | 0.522 | 37.47 | <0.001 |
| Radiotherapeutic Oncology | 0 | 10.00 | 0.251 | 0 | - |
| Pediatrics | 0 | 0 | - | 0 | - |
| Gastroenterology | 157.11 | 147.26 | 0.695 | 66.92 | <0.001 |
| Neurology | 12.93 | 9.22 | 0.709 | 9.00 | 0.689 |
| Endocrinology | 11.79 | 23.92 | 0.295 | 3.47 | 0.259 |
| Intensive Care | 317.46 | 727.27 | 0.485 | 688.07 | 0.491 |
| Preventive Medicine | 99.75 | 290.82 | 0.161 | 101.69 | 0.982 |
| Immunology | 0 | 0 | - | 0 | - |
| Internal Medicine | 87.03 | 133.63 | 0.220 | 131.65 | 0.227 |
| Medical Oncology | 20.68 | 31.02 | 0.364 | 34.09 | 0.277 |
| Pneumonology | 0 | 3.62 | 0.346 | 3.14 | 0.380 |
| Allergy | 5.54 | 0 | 0.290 | 4.92 | 0.934 |
| Nephrology | 109.48 | 114.32 | 0.893 | 166.34 | 0.169 |
| Psychiatry | 16.84 | 17.26 | 0.980 | 41.31 | 0.309 |
| Cardiology | 1.93 | 3.49 | 0.625 | 5.84 | 0.314 |
| Rheumatology | 35.22 | 39.40 | 0.853 | 65.41 | 0.243 |
| **Surgical** | 4.68 | 4.86 | 0.921 | 7.50 | 0.183 |
| Anesthesiology | 22.39 | 14.95 | 0.656 | 32.45 | 0.625 |
| Ophthalmology | 0 | 0 | - | 0 | - |
| Gynecology | 20.41 | 7.87 | 0.144 | 10.95 | 0.301 |
| Vascular Surgery | 0 | 17.58 | 0.136 | 11.86 | 0.221 |
| Dermatology | 0 | 23.51 | 0.008 | 35.17 | 0.001 |
| Plastic Surgery | 0 | 0 | - | 0 | - |
| Thoracic Surgery | 0 | 0 | - | 0 | - |
| Maxillofacial Surgery | 0 | 0 | - | 0 | - |
| General Surgery | 0 | 0 | - | 6.43 | 0.303 |
| Neurosurgery | 0 | 0 | - | 0 | - |
| Otorhinolaryngology | 4.82 | 0 | 0.276 | 0 | 0.334 |
| Traumatology | 2.87 | 0 | 0.295 | 0 | 0.331 |
| Cardiac Surgery | 0 | 0 | - | 0 | - |
| Urology | 0 | 0 | - | 4.70 | 0.333 |

* p-value long-term vs. before training

***Table S5c.*** *New HCV active infection (positive antigen) rate per 10^5^ attended patients per department in pre-, post-session, and long-term periods.*

| Departments | New HCV positive antigen/10^5^ attended patients | | | | |
| --- | --- | --- | --- | --- | --- |
|  | Before Training | After  Training | *p*-value | Long-term | *p*-value* |
| **Globally: Medical + Surgical** | 6.85 | 7.51 | 0.646 | 3.72 | 0.012 |
| **Medical** | 11.02 | 12.21 | 0.616 | 5.97 | 0.013 |
| Emergency Department | 0 | 1.34 | 0.304 | 4.01 | 0.075 |
| Radiotherapeutic Oncology | 0 | 0 | - | 0 | - |
| Pediatrics | 0 | 0 | - | 0 | - |
| Gastroenterology | 79.60 | 68.44 | 0.525 | 33.46 | 0.003 |
| Neurology | 4.31 | 0 | 0.334 | 0 | 0.328 |
| Endocrinology | 0 | 10.25 | 0.106 | 0 | 1.000 |
| Intensive Care | 0 | 0 | - | 0 | - |
| Preventive Medicine | 0 | 0 | - | 0 | - |
| Immunology | 0 | 0 | - | 0 | - |
| Internal Medicine | 31.08 | 49.23 | 0.428 | 6.27 | 0.105 |
| Medical Oncology | 2.59 | 11.93 | 0.125 | 9.30 | 0.236 |
| Pneumonology | 0 | 0 | - | 0 | - |
| Allergy | 0 | 0 | - | 0 | - |
| Nephrology | 0 | 5.44 | 0.344 | 0 | - |
| Psychiatry | 0 | 0 | - | 13.77 | 0.201 |
| Cardiology | 0 | 0 | - | 0 | - |
| Rheumatology | 0 | 6.57 | 0.334 | 11.89 | 0.194 |
| **Surgical** | 0.72 | 0.69 | 0.971 | 0 | 0.177 |
| Anesthesiology | 7.46 | 7.47 | 0.999 | 0 | 0.337 |
| Ophthalmology | 0 | 0 | - | 0 | - |
| Gynecology | 0 | 0 | - | 0 | - |
| Vascular Surgery | 0 | 0 | - | 0 | - |
| Dermatology | 0 | 3.36 | 0.319 | 0 | - |
| Plastic Surgery | 0 | 0 | - | 0 | - |
| Thoracic Surgery | 0 | 0 | - | 0 | - |
| Maxillofacial Surgery | 0 | 0 | - | 0 | - |
| General Surgery | 0 | 0 | - | 0 | - |
| Neurosurgery | 0 | 0 | - | 0 | - |
| Otorhinolaryngology | 0 | 0 | - | 0 | - |
| Traumatology | 2.87 | 0 | 0.295 | 0 | 0.331 |
| Cardiac Surgery | 0 | 0 | - | 0 | - |
| Urology | 0 | 0 | - | 0 | - |

* p-value long-term vs. before training
